# Notes on Preparations for the Sick

**Published:** 1898-06

**Authors:** 


### Notes on Preparations for the Sick.

---

**Tabloids: Thyroid Colloid; Lactophenin; Colchicine Salicylate; Chemical Food; Cotarnin Hydrochloride (Stypticin).**—BURROUGHS, WELLCOME & Co., London.—We have never had reason to think that drugs in tabloid form were less active than when given as powder, or in pill or cachet. The 4-grain **Lactophenin** tabloid is likely to be a very efficient antipyretic, and the **Colchicine** (gr. $\frac{1}{23}$) tabloid a very convenient form for the waistcoat pocket of the gouty man. The **Chemical Food** tabloids are sugar coated and quite inviting. The **Cotarnin** tabloids are prepared from narcotin, one of the opium alkaloids, and they are used in the treatment of uterine hemorrhage. They are found most useful in cases of uterine subinvolution, and in endometritis accompanied with menorrhagia. Gottschalk has made numerous experiments with stypticin and speaks highly of its value as a hæmostatic. The tabloids each contain $\frac{3}{4}$ grain of the drug, and form a handy method of administering it.

---

"**Ambrosia.**"—EVANS, GADD & Co., Exeter.—The idea of combining Devonshire cream with an active extract of malt was one which the cod liver oil taking patient was not slow in appreciating. The compound called "Ambrosia" contains 75 parts of the Exonia brand of malt extract, with 25 parts of pure cream. Its price has been its only disadvantage, and now that this objection has been removed it should have an increasing popularity amongst others than the gods. We have found that there is usually a decided gain in weight when the drug is taken in fair quantity.

---

**Sterilised Milk.**—I. F. ARROWSMITH, Clifton.—The value and importance of sterilised milk is now well known, and we are glad to see that a local firm has had the enterprise to procure the necessary machinery for rendering milk sterile and keeping it so. By the courtesy of the manager we were able to see the whole process from start to finish, and we were much struck by the perfect cleanly and scientific manner in which the heating and storing was done. The milk has a slight saccharine flavour, but is of the same nutritive value as ordinary milk, and has the advantage that it can be kept in the hermetically sealed bottles for any length of time. We expect and hope for a considerable demand for this pure milk. On account of the evils that have come to us through milk, its sterilisation is an important matter and one to be very seriously considered.

**Cream Milk for Infants.**—AYLESBURY DAIRY COMPANY, London.—As its name implies, this milk is specially rich in cream, a constituent so important in the feeding of infants. It deserves, and we have no doubt receives, extensive patronage.

---

**Nestlé's Condensed Swiss Milk.**—HENRI NESTLÉ, London.—This is so well known to the profession, that further confirmation of its value is hardly necessary. Its extensive use is fully warranted by its purity and constant accuracy of composition.

---

**Cabot Bread and Biscuits.**—HUMPHRIES & BOBBETT, Bristol.—A sample of this bread, now so well known in Bristol, has been forwarded to us, and we find it exceedingly agreeable eating and very readily digested. It is, further, pleasant to the taste, not being too moist or gritty. We can recommend it as a thorough good article of home production, although, not knowing the processes through which the flour has passed, we are unable to say in what its special virtue consists. **Biscuits** with the same distinctive appellation have also been sent. These we found very palatable.

---

**Vimbos.**—VIMBOS, LIMITED, London.—This is another fluid meat extract, shewn by Dr. Stevenson Macadam's analysis to contain nitrogenous organic matter amounting to over 50 per cent. It is a very viscid fluid, which, diluted to twenty times its bulk, gives a very abundant precipitate of albumen when boiled or treated with picric acid. There is nothing objectionable in either odour or taste, and we can quite accept the statement that it must be far more nourishing than ordinary beef tea. It makes excellent beef tea, or it may be used as a paste upon bread. "In all respects Vimbos is a fluid beef of the highest order."

---

**Bovril Stamnoids.**—BOVRIL LIMITED, London.—These tabloids are described as "a lunch in the waistcoat pocket." They are not unpleasant to the palate, and they must contain an appreciable amount of easily assimilated nourishment in the smallest possible bulk, suitable for cyclists and others who want food by the way at times and places where set meals cannot be procured.

---

**Milk Chocolate.**—CADBURY BROS., Bourneville, Birmingham.—This very palatable preparation is suitable for carrying in the pocket, and must provide a more sustaining and nutritious food than the ordinary chocolates without the milk. Cadbury's preparations are well known to be genuine, and to be free from adulteration with all harmful drugs.

**Teething Pad.**—MELLIN'S FOOD, LTD., London.—This is a cruciform porcelain ornament attached to a cord for hanging round the neck of the infant--two arms of the cross are covered with India-rubber. The apparatus appears to be designed in order that infant and nurse may have continually before them a reminder that Mellin's Food is suitable to the infant, and Mellin's Food Biscuits after teething. We have found that these biscuits are most acceptable to the convalescing typhoid patient of all ages.

---

Claiming to make refraction work easy for general practice, Mr. F. Davidson of London has introduced to our notice his complete sight test, which consists of an optometer, somewhat similar to that of Doyne, and of a specially constructed stiffened card, bearing, besides the usual Snellen's types, a graduated series of parallel lines, for estimating astigmatism, a set of colours, and a scale for measuring strabismus, etc. Mr. Davidson has also written a concise booklet bearing on these subjects.

The optometer is composed essentially of a number of concave and convex-spherical lenses, fitted into the margins of a pair of independently revolving mahogany discs, the whole being mounted on an adjustable brass stand. By a simple arrangement, these discs can be so adjusted that they correspond to, and indicate, the inter-pupillary distance; and by their rotation, successive lenses are brought in front of each of the patient's eyes, until the maximum visual acuity is attained. The instrument can be used for the retinoscopic as well as for the subjective method of examination, and by its means errors of refraction of from .25 degree to 19 degrees or 20 degrees can be rapidly arrived at; it is strongly made and well-finished, whilst it is impossible for the lenses to get lost or out of place.

We gladly welcome any means by which the practitioner may acquire sufficient confidence to test the sight of his patients himself, instead of placing them in the hands of the "consulting oculistic optician."

